# A lab-on-a-chip for hypoxic patch clamp measurements combined with optical tweezers and spectroscopy- first investigations of single biological cells

**DOI:** 10.1186/s12938-015-0024-6

**Published:** 2015-04-18

**Authors:** Ahmed Alrifaiy, Johan Borg, Olof A Lindahl, Kerstin Ramser

**Affiliations:** Institute of Neuroscience and Physiology, Section of physiology, Gothenburg University - Sahlgrenska Academy, Göteborg, 405 30 Sweden; Department of Computer Science, Electrical and Space Engineering, Luleå University of Technology, Luleå, 971 87 Sweden; CMTF, Centre for Biomedical Engineering and Physics, Luleå and Umeå, Sweden; Department of Radiation Sciences, Biomedical Engineering, Umeå, 901 87 Sweden; Department of Engineering Sciences and Mathematics, Luleå University of Technology, Luleå, 971 87 Sweden

**Keywords:** Microfluidic system, Hypoxia, Patch clamp, Optical tweezers, Absorption spectroscopy, Oxygen sensor

## Abstract

The response and the reaction of the brain system to hypoxia is a vital research subject that requires special instrumentation. With this research subject in focus, a new multifunctional lab-on-a-chip (LOC) system with control over the oxygen content for studies on biological cells was developed. The chip was designed to incorporate the patch clamp technique, optical tweezers and absorption spectroscopy. The performance of the LOC was tested by a series of experiments. The oxygen content within the channels of the LOC was monitored by an oxygen sensor and verified by simultaneously studying the oxygenation state of chicken red blood cells (RBCs) with absorption spectra. The chicken RBCs were manipulated optically and steered in three dimensions towards a patch-clamp micropipette in a closed microfluidic channel. The oxygen level within the channels could be changed from a normoxic value of 18% O _2_ to an anoxic value of 0.0-0.5% O _2_. A time series of 3 experiments were performed, showing that the spectral transfer from the oxygenated to the deoxygenated state occurred after about 227 ± 1 s and a fully developed deoxygenated spectrum was observed after 298 ± 1 s, a mean value of 3 experiments. The tightness of the chamber to oxygen diffusion was verified by stopping the flow into the channel system while continuously recording absorption spectra showing an unchanged deoxygenated state during 5400 ± 2 s. A transfer of the oxygenated absorption spectra was achieved after 426 ± 1 s when exposing the cell to normoxic buffer. This showed the long time viability of the investigated cells. Successful patching and sealing were established on a trapped RBC and the whole-cell access (Ra) and membrane (Rm) resistances were measured to be 5.033 ± 0.412 M *Ω* and 889.7 ± 1.74 M *Ω* respectively.

## Introduction

The patch clamp technique [1] is a significant tool in electrophysiology for high resolution investigations of the ionic current activities through the membrane of living biological cells. The technique provides critical insight into wide ranging applications in the fields of neuroscience, biology, pharmacology, and many other related biomedical research disciplines. The electrophysiological activity of individual biological cells are measured and analyzed by high-resolution current-, and voltage recordings of whole cells, through single ion channels or removed cellular patches. In patch clamp experiments the biological cells are exposed to fast changing environmental stimuli created by an open perfusion system. The electrical signaling activity across the plasma membrane of the cell is recorded through an electrode placed in the extracellular environment close to the cell and another recording electrode that is placed in the micro-pipette of borosilicate glass. The tip of the pipette is patched to the cell membrane to perform recordings on the cell under varying stimuli. Patch clamp is one of the most challenging methods in daily laboratory work. The technique needs highly sensitive equipment and operators must have high experimental skills.

Many attempts have been made to modify the traditional patch clamp technique [2]. Planar patch clamp [3] is the most reported one; it bases on planar microfluidic chip substrates of 1-2 micro-sized holes for capturing the cells from suspension by means of suction. The cells are sealed to the substrate by applying suction pulses or pore-forming compounds for whole cell and perforated patch recordings respectively. The benefits are the multiple cell recordings by arrays of apertures, lower cost and data throughput. The main challenges associated with planar platforms are the lower data quality outputs by a reduced electrical seal between the recording substrate and the cellular membrane [4]. Another challenge is that the G *Ω*-seal created here is formed as cell membrane-substrate resistive interaction, which requires improvements for measurements associated with low-noise, high-quality recordings and high temporal resolution [5].

Even though the planar platforms overcome some key challenges, the conventional patch clamp technique still enables the best temporal resolution, voltage control and direct measurements. The high level of flexibility and experimental possibilities for environmental exchange are valuable, necessary and usually optimized related to the nature of experiments.

Most electrophysiological studies on oxygen deprivation, e.g. stroke, are carried out either by inducing a chemical oxygen shortage or by flushing cells or tissue with oxygen free solutions in open systems. In both ways, the effective oxygen content in-situ cannot be sufficiently controlled due to the diffusion of ambient oxygen and often the oxygen content is close to the one present *in vivo* (about 6%) [6-9]. However, those estimations are away from providing an accurate value of the oxygenation states of the investigated cells. Many studies demonstrate the need of environmentally controlled LOC systems for biological applications [10]. LOC devices offer important features for precise control of the amount and the dynamics of reagents within micro-sized fluidic channels connected to reservoirs, inlets and outlets. Those systems are usually fabricated in optically transparent materials [11,12], suitable for most types of optical microscopes and can be combined with other optical techniques. LOC devices, as essential tools for spatial and temporal control of cells and fluids on the micro-sized level, have been used for precisely controlled oxygen micro-environmental conditions such as gas mixing and chemical de-oxygenation [13-20]. For instance, PolyDiMethylSiloxane (PDMS)-based LOC, established on exchangeable passive diffusion of oxygen and nitrogen, have been used in hypoxic cell investigation to control oxygen concentration gradients [21-23]. However, the regulation and the generation of oxygen gradients through electrolysis in LOC for various cell assays are challenging due to the complexity to incorporate gas sources, device manufacturing and the possible impact on the well-being of the investigated cells [24-26]. Other studies that rely on oxygen content variations during cell culturing in LOCs include cell-based assays [27-31], bioreactors [32-34], and tissue engineering [35]. Furthermore, LOC based oxygenation methods have been reported to use and treat the medium as the oxygen source [36-39]. However, the passive diffusion method is not sufficient for the demand of dense cell cultures due to the low solubility of oxygen in aqueous solutions and hence the long term diffusion time. Additionally, the devices are mainly produced of PDMS having a high oxygen permeability [40]. Hence, full anoxic conditions cannot be established.

Innovative design is required to modify the conventional patch-clamp technique in which a micropipette is steered in three dimensions (3D) for precise attachment to the cell membrane for patching in a closed system. The promising approach presented here is to achieve control of the gaseous surroundings of the investigated cells by a gas-tight LOC system of Plexiglas with an integrated pipette for patch clamp experiments. The positioning of an individual cell in 3D within the closed micro-channel system is enabled by optical tweezers [39], an excellent tool to trap and manipulate biological cells. Optical tweezers use light radiation forces to trap and steer small dielectric objects in three dimensions [41]. Experimentally, a stable optical trap is achieved by focusing a laser beam strongly through a high-numerical aperture (NA) microscope objective onto the sample. The trapped object is usually manipulated by moving the trap and/or the sample stage to the desired position.

Previously, we presented a LOC that had the capability of steering a biological cell towards a micropipette. However, the system allowed only for chemical oxygen deprivation by adding natrium dithionte and no strong cell-membrane seals, necessary for patch-clamp experiments, could be performed [42].

The aim of this study was to design, develop and manufacture a LOC device for electrophysiological and spectroscopic studies on viable optically trapped biological cells demanding complete oxygen level control. The oxygen level was monitored by an integrated oxygen sensor.

We present a prototype of a new developed gas-tight multifunctional LOC system for hypoxic investigations on biological cells. The PolyMethylMethAcrylate (PMMA)-based LOC was manufactured by using Computer Numerical Control (CNC) machine for simplicity and to enable quick reproducibility in shorter time and lower cost. The gas-tight efficiency and flexibility of LOC were facilitated by a new design of microfluidic channels, a novel fastening technique and smart Micro-to-Macro interconnections between the microfluidic channels and the exterior techniques, as compared to our previous work [42]. The viability of the cells was improved by efficient cell transport into the LOC using shorter transport pathways, effective design of the channel system and enhanced fluid flow. A new concept was developed to insert the patch clamp pipette to the LOC by replacing the hollow screw, used in our previous study [42]. The new method enabled a precise steering of the pipette into the LOC by an attached single-axel micrometer differential drive, fitted together with the LOC as a platform, placed on the stage of the microscope.

## Materials and methods

### Biological samples and experimental buffer solutions

RBCs from chicken (Fitzgerald Industries International, USA) were used as a sample model in this study. The cells were pre-treated by washing, brief exposure to glutaraldehyde buffer solution and exhaustive washing in saline solution before suspension in saline solution with 0.1% sodium azide. The cells were then incubated at 4°C to ensure a steady state related to ion and water contents before experimental treatment. The sample consisted of 0.05 ml RBCs diluted in 2 ml Extracellular (EC) solution at pH 7.0 and 22°C.

Extracellular physiological solution (EC) of 150 mM KCl, 10 mM CaCl _2_, 5 mM MgCl _2_, 1 mM MES was titrated to pH 7.0 with Tris base (Sigma Aldrich, Germany) and used as bath medium for flushing the cells.

Intracellular solution (IC) containing 195 mM KCl, 1 mM EGTA, 0.15 mM CaCl _2_, 4 mM MgCl _2_, 4 mM ATP, titrated to pH 7.0 with KOH was used as pipette-filling solution. The deoxygenated EC solution was prepared by purging the solution with nitrogen gas (AGA AB, Sweden). Prior to the experiment, the nitrogen flow rate was controlled and the rate of oxygen level was measured continuously by an oxygen sensor system of a fiber optics probe (FOXY, AL3000, Ocean Optics, USA) connected through MultiFrequency Phase Fluorometer (MFPF, ocean optics, USA). All solutions were prepared in bottles and sucked into the syringes of a peristaltic pump system (HPLC, K-501, Germany) and a programmable pump system (neMESYS, Cetoni, Germany).

### Experimental set-up

**Figure 1:**
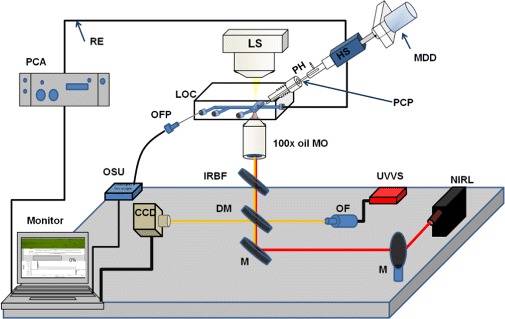
A schematic figure of the experimental setup consisting of 1) LOC; gas-tight lab-on-a-chip, with an integrated micropipette, combined with 2) optical tweezers consisting of an (NIRL) NIR-laser, (M) mirrors, (DM) dichroic mirror and an (IRBF) IR blocking filter (to block the IR laser to the CCD), 3) (UVVS) UV-Vis absorption spectrometer including an integrated (OF) optical fiber, 4) CCD camera of the microscope (to monitor the trapping dynamics and the pipette cell patch), 5) (OSU) oxygen sensor unit connected to (OFP) optical Foxy Probe inserted into inlet of the LOC), 6) patch clamp set-up consisting of (MDD) micromanipulator connected to (HS) patch clamp head stage with (PH) pipette holder for insertion of the (PCP) patch clamp pipette through and adapter into the chip, (RE) recording electrode connected to (PCA) patch clamp amplifier through the (HS) and the reference electrode connected between an inlet of the LOC and (HS).

The experimental system was based on a combination of LOC, optical tweezers, patch-clamp, absorption spectroscopy and oxygen sensing in a multifunctional system. The experimental setup was built on an inverted microscope (IX71, Olympus, Japan). The trapping laser was guided into the microscope through the upper rear port while the absorption spectrometer was fitted at the side port. The microscope and supplementary devices were ergonomically placed on a vibration-free optical table (TMC, USA), as presented briefly below in Figure 1.

#### Optical tweezers

Optical tweezers [40] were built upon an near infrared (NIR)-diode laser (Renishaw, UK), with a square beam profile (size 5 mm) operating at 830 nm ± 1 nm with an average power of 300 mW ± 30 mW. The power at the sample was about 148 mW. The wavelength of the laser (830 nm) was chosen to minimize heating and photodamage of the sample [43]. All equipment was mounted on a XYZ-translation stage (Thorlabs Inc., USA) to ensure precise alignment. The beam of the trapping laser was steered through a system of mirrors (Thorlabs Inc., USA) into the microscope oil immersion objective (100 ×, 1.4 NA, Olympus, Japan) through a dichroic mirror (Chroma Technology, USA). The intensity profile of the laser beam overfilled the back focal plane of the objective; hence no beam expansion was necessary. The optical trap, situated at the focal distance of the microscope objective, was aligned to the centre of the field-of-view of the Charge Coupled Device (CCD) camera of the microscope.

#### Structural design of LOC and integrations

**Figure 2:**
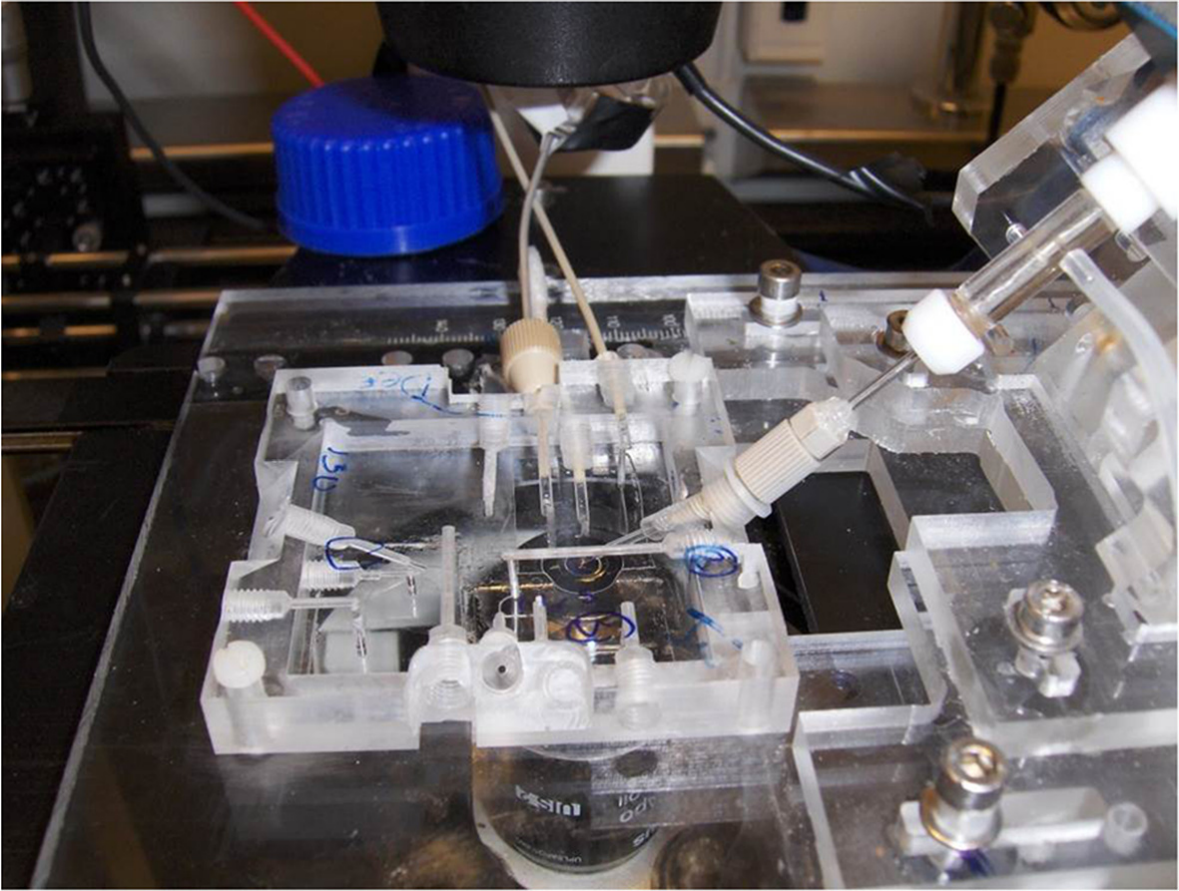
Photograph of the PMMA based LOC system placed on an inverted microscope.

A new prototype of LOC was developed, see Figure 2, following the previously presented concept [42], by using (CNC) machine and manual drilling to create PMMA based LOC system. The microfluidic structures were designed using a software program (Auto-CAD, Autodesk, USA) and transformed to a face of a square PMMA slab by CNC milling. For the insertion of the patch clamp pipette into the LOC, a diagonal hole (at 45 degrees) was drilled manually, starting from the upper edge of the LOC to point towards the intersection zone, in which the biological cell was investigated within the channel system of the LOC. The air tightness around the drilled hole was guaranteed by using gas-tight fittings (Polyether ether ketone (PEEK), ScanTec, Sweden) of diameters matching the outer diameter of the used patch clamp micropipette.

**Figure 3:**
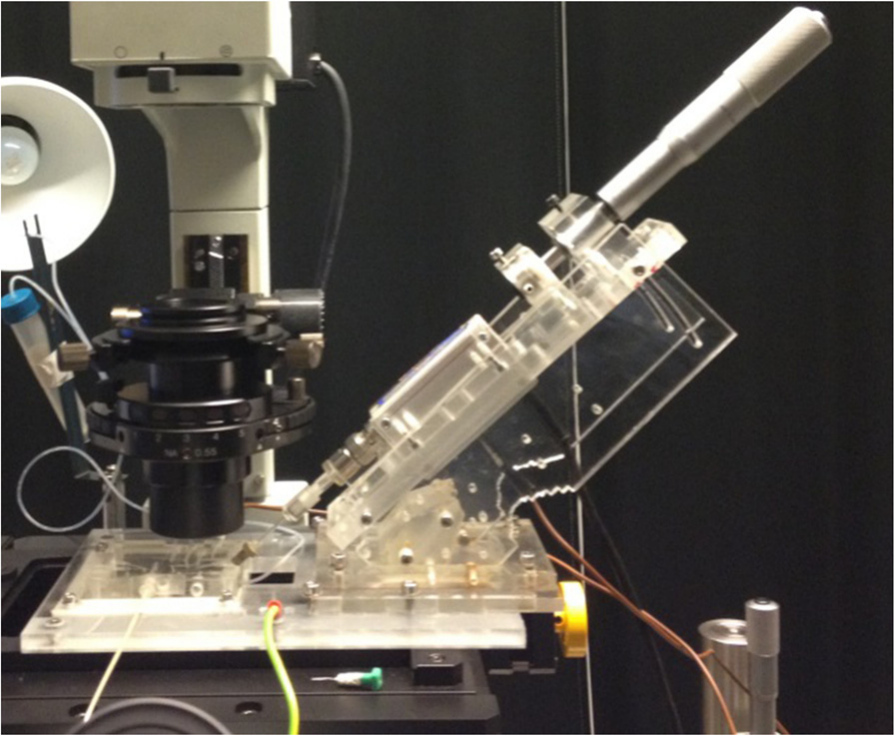
A platform of the multifunctional LOC system combined with patch-clamp, built on an inverted microscope.

The surface of the PMMA slab was milled flat and parallel to the CNC machine coordinate system using a 6 mm carbide end-mill. A 2-flute 150 *μ*m carbide end-mill (LPKF 115832, Germany) operating at 25 kRPM and with a feed-rate of 2 mm/s was then used to mill the microfluidic channels. This change of tooling was necessary due to the excessively slow material removal when using the 150 *μ*m tool. To manufacture channels with correct depth, a calibration step was introduced in which a set of progressively deeper trial channels were milled close to the final location of the microfluidic channels. The vertical location of the tool relative to the surface of the PMMA could then be determined accurately by counting the number of lines milled into the PMMA as any milling at all was readily apparent to the naked eye. The size of the final channels was 150 × 40 *μ*m (width × height). Compared to [42], the LOC was equipped with smart lateral interconnections between the micro-size channels and the external devices. The inlets and outlet, adjacent to the microfluidic channels, were manually drilled, as side-holes (*ϕ* = 1 mm), through the outer side-edge of the LOC. The external ends of the holes were then threaded internally to enable gas-tight connections of the channel system to the pump systems through tubing, screws and fittings. This ensured the gas-tight functionality and flexibility. The microfluidic channels were closed with a cover glass of 24 × 36 × 0.055 mm (Gerhard Menzal, Germany) using Ultraviolet (UV)-curable adhesive material (EPO-TEK OG603, Epoxy Technology, USA). Three inlets and the waste outlet of the final chip were connected to two pump systems, by gas-tight (PEEK) tubing, for an independent infusion of RBCs and EC buffer solutions. An inlet was designed especially for the insertion of an oxygen sensing probe close to the interaction zone of the channels, i.e., the area in which the RBCs were investigated. Another inlet was designed specifically to introduce the reference electrode of the patch clamp technique into the microfluidic channels of the LOC. The concept of introducing the patch clamp pipette to the LOC by a hollow screw in a previous study [42] was replaced by a smart design in which the pipette was positioned and steered into the LOC by an attached single-axel micrometer differential drive. The drive was fitted together with the LOC as a platform and placed on the stage of the microscope. The stable platform was important to enable a precise micro-poisoning of the tip of the pipette in the desired patching position within the channel (Figure 3).

#### Optical absorbance measurement’s instrumentation

The oxygenated and deoxygenated conditions of the trapped RBC were monitored by an integrated spectrometer for absorption spectroscopy in the visible region (Ocean Optics, HR4000, USA). The visible light from the microscope passing through the sample and the dichroic mirror was split by a beam splitter; a fraction of 20% of light was guided to the camera on the left hand side of the microscope and 80% was guided to the right hand side port of the microscope to the spectrometer, see Figure 1. The optical fiber, with a core size diameter of 50 *μ*m, was aligned precisely onto the center of the right-hand side-port of the microscope to collect the transmitted light. The absorption spectra were binomially smoothed by a data-analyzing software program (IGOR Pro, USA). The region from 520 - 600 nm was used to study the typical spectral peaks of the Q-bands of the oxygenated and deoxygenated states of haemoglobin (Hb) [44]. The built in function “Binomial smoothing” as a Gaussian filter was applied to reduce the variability of data as well as to present clear and noise-free spectra [45]. We were careful to ensure that the peaks at 540 nm, 575 nm and 553 nm were not changed in both cases before and after using the smoothing operation.

#### Patch clamp

Patch clamp [1] was used to register the electrical activity across the plasma membrane of an optically trapped chicken RBC. An electrode was placed in one inlet of the LOC and a recording electrode was placed in the micro-pipette to record the electric signals. Signals were recorded using an amplifier (EPC-7, HEKA elektronik, Germany), a Digidata 1200 interface and software program pClamp 7 (Axon Instruments, Union City, CA, USA). Patch clamp pipettes were pulled from borosilicate glass capillaries of 1.5 mm (OD), 1.16 mm (ID) (PG150T-10, Harvard apparatus, USA) in two steps using a vertical Needle/Pipette Puller (David Kopf Instruments, Model 750, Tujunga, CA, USA). First, the capillary was thinned over a length of 7-10 mm to obtain a minimum diameter of 150 *μ*m. The capillary was then recentred related to the heating filament of the puller. In the second step the thinned pipette was pulled until break to produce two pipettes with tips of about 1-2 *μ*m. For this experimental purpose, the puller was adjusted to produce one pipette of good quality to fill out the required properties, i.e., tip’s diameter and taper’s length to be used for experimentation.

#### The oxygen sensor system

The oxygen content was measured through a fibre optic oxygen sensor probe, (FOXY probe, ocean optics, USA), connected to a MultiFrequency Phase Fluorometer (Ocean Optics, Florida, USA). The oxygen sensor was pre-calibrated by curves generated from standards concentration values of O _2_ dissolved in anoxic and normoxic solutions. The second order polynomial algorithm was used for better curve fitting and for accurate oxygen measurements in a broad oxygen concentration range. The continuous visualization of the O _2_-concentration values were achieved by a software program (OOISensors Oxygen Measurement Software, Ocean Optics, USA) installed on a personal computer (PC) connected to the oxygen sensor system, see Figure 1 above.

## Experimental results and discussions

In a first step, the LOC was manufactured as described in the section of Materials and Methods. To enable the measurements under full control of the environment, a novel approach in the fabrication process was introduced to achieve good quality fastening of the LOC by using the capillary effect. This was performed by using a rectangular cover glass that was attached to the LOC with a UV curable adhesive material, characterized with low viscosity, high optical transparency and high biocompatibility (USP Class VI).

**Figure 4:**
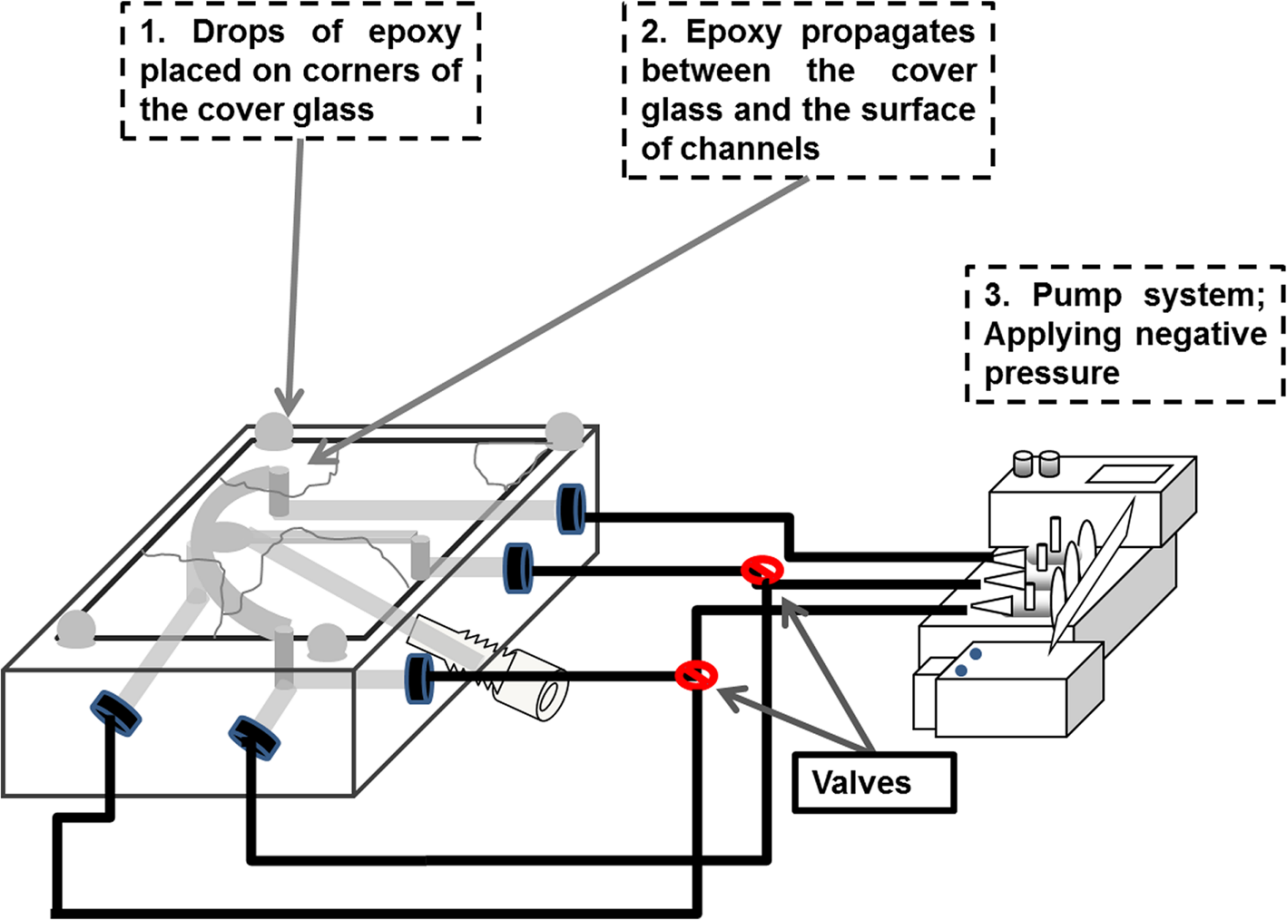
The CNC machined microfluidic channels on PMMA material were connected to the pump systems by manually drilled holes extended to the side faces of the microfluidic chip. The sealing was performed by 1) a cover glass placed on the channel surface, 2) drops of adhesive epoxy placed on the corners of the attached cover glass and 3) negative pressure applied through the side-holes joined to the channels.

**Figure 5:**
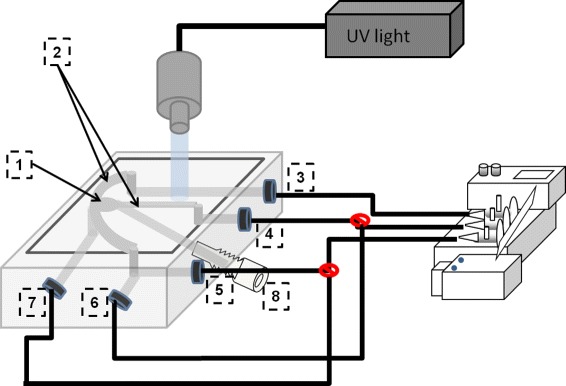
UV light curing of the epoxy while applying negative pressure into the channels. The figure shows 1) interaction (patching) zone, 2) microfluidic channels, 3) inlet for normoxic EC solution, 4) inlets for the biological cells, 5) inlet for the anoxic EC solution, 6) inlet for the probe of the oxygen sensor, 7) outlet and 8) the diagonal hole to insert the patch-clamp pipette through gas-tight adapter.

As seen in Figures 4 and 5 below, the etched side of the LOC was placed face up. A clean cover glass was positioned to cover the etched channel pattern and to enclose the channels. The adhesive epoxy was carefully applied drop-wise to the edges of the cover glass. The adhesive epoxy was then spread slowly by capillary action to form a thin layer between the two surfaces. After 4.0 min of full adhesive coverage, a negative pressure was applied through the inlets of the LOC using the pump system. This guaranteed that the cover glass was well-attached to close the channels and to remove epoxy that might have propagated into the channels. Throughout the previous step, the final step was to cure the adhesive epoxy by UV light. Prior to the experiment, the functionality of the closed channels was validated to ensure the perfect attaching of the cover glass and that there was no contamination in the channels from the epoxy. This was achieved by investigation of the channels; optically under microscope and by applying fluid flows, generated by a pump system, through the microfluidic channels.

The gas-tight microfluidic chip including the integrated micropipette was positioned on the microscope stage and connected to the pump systems for the insertion of RBCs and for the flow of the solutions of different oxygen contents, see Figure 3.

The micropipette was carefully inserted, through the diagonal hole in the LOC, into the intersection-zone within the microfluidic channel and monitored visually. The selected RBCs were introduced to the microfluidic channel system in a low flow rate (0.1 *μ*l/s) to enable the monitoring and selection of an RBC for experiments. The RBCs were trapped by optical tweezers and optically steered within the microfluidic channels towards the interaction-zone, while the trapping dynamics were recorded in real time with the CCD-camera of the microscope. The optical trapping and steering of RBCs has been presented previously in ref [42]. The LOC including the integrated micropipette was precisely moved in 3D related to the fixed trap. The trapped RBC was steered to the interaction zone of the channel where the tip of the patch clamp micropipette was located. The micropipette was moved exactly towards the cell to place the tip onto the membrane of the cell. This experiment was performed for 13 times prior to the patch clamp experiments to ensure the functionality of the design.

To verify the gas-tight functionality of the microfluidic chip, the oxygen content was measured by an oxygen sensor while absorption spectra were taken in the visible region. The oxygen sensor measured values from 18% oxygen in normal EC solution that gradually declined down to 0-0.5% oxygen when deoxygenated-buffer solution (0% O _2_) was purged into the channel system. The Experiments were performed under real physiological oxygen transition compared to our earlier study [42] in which hypoxia was generated chemically by adding natrium dithionite [46].

**Figure 6:**
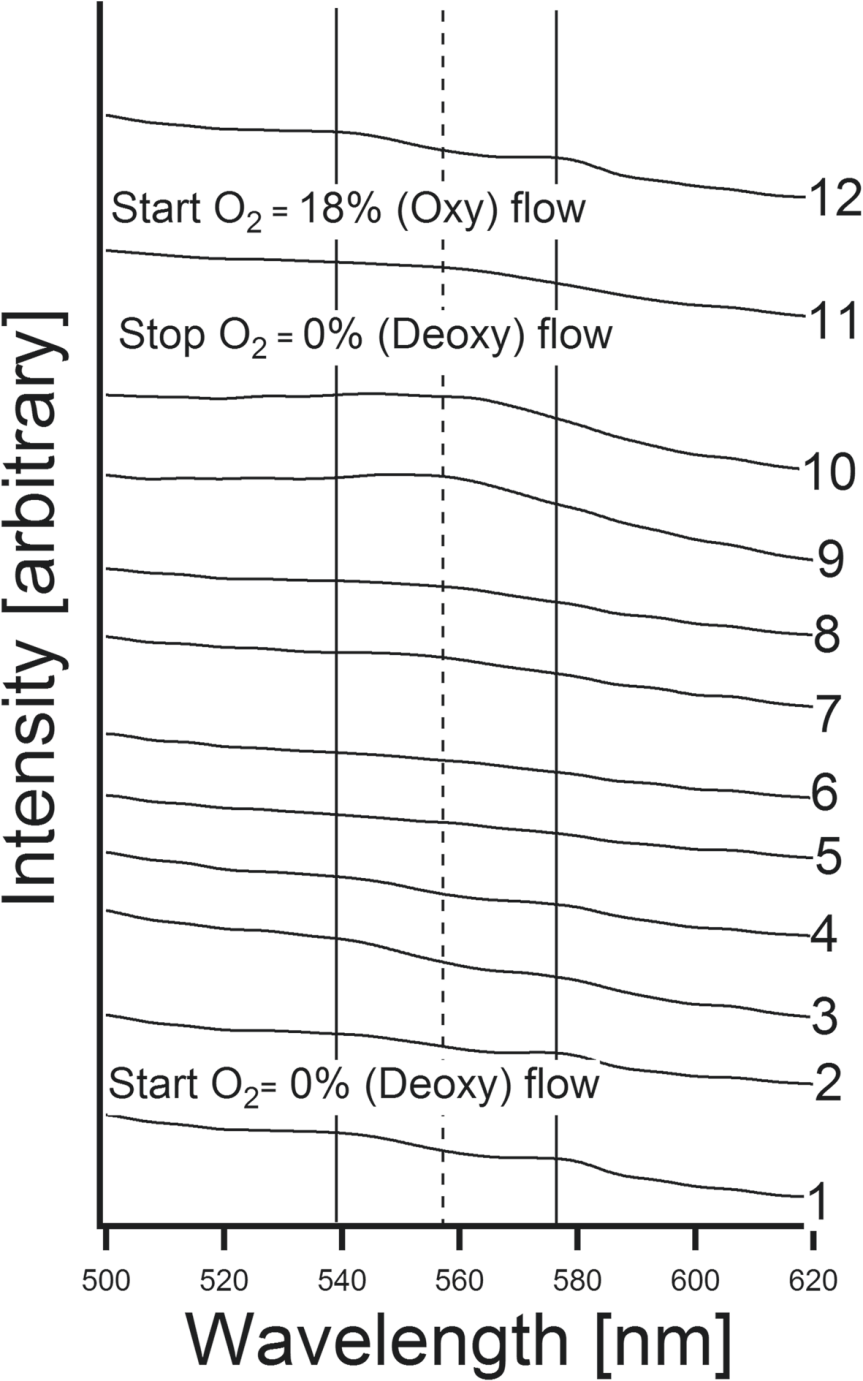
Absorption spectra from average of four optically trapped chicken RBCs. Peaks at the straight lines indicate oxygenated state, at the dotted line indicates deoxygenated state. At time (t = 0 s) we see the oxygenated state(spectra No. 1). After 289 s of flow with buffer EC deoxygenated solution (0% O _2_) the deoxygenated state was reached(spectra No. 10). After that the flow of (0% O _2_) was stopped to see whether oxygen could diffuse into the LOC. After 5400s the deoxygenated state was still shown (Spectra No. 11) and latterly, a flow of oxygenated EC buffer solution (18% O _2_) was started. After 428 s the oxygenated state was reached again (Spectra No. 12).

The absorption spectroscopic measurements in the visible region were started by acquiring and monitoring the absorption spectra of the trapped cells under exposure of normal EC solution with 18% oxygen. The experiment was repeated three times. The environment of the investigated cell was then changed by purging a (0% O _2_) EC deoxygenated solution. The results showed the transformation of the absorption spectra of Hb in the RBCs from the oxygenated to the deoxygenated states. The monitored oxygen contents and the corresponding time series are presented for one experiment, see Figure 6 and Table 1. At the beginning of the experiment (t = 0 s), the absorption spectrum was acquired in the oxygenated state (17.83% O _2_). The spectrum showed one strong peak at 540 nm and another peak at 575 nm, which is equivalent to the reported spectra of Hb in red blood cells from chicken [44]. The trapped RBCs were then deoxygenated by a flow of (0-0.5% O _2_) EC buffer solution and a time series of spectra showed the gradual transformation from the oxygenated to the deoxygenated state. The fully developed absorption spectrum of the RBC in the deoxygenated state showed the typical peak at 553 nm [44]. To prove the gas-tight efficiency of the LOC, the flow of (0% O _2_) buffer solution was stopped to see if any oxygen could diffuse into the microfluidic channel. The acquired absorption spectra of the trapped chicken RBCs showed no change of the deoxygenation state during 5400 s, as seen in Figure 6. This shows that the LOC provided a full control over the oxygen content. The back transform to the oxygenated state was acquired by exposing the trapped cells to normoxic EC solution (18% O _2_), showing a gradual back transformation of the spectra from the deoxygenated to the oxygenating state, which verified that the investigated RBCs were viable for more than 97 minutes. The values of oxygen level, measured by the oxygen sensor within the microfluidic chip scheduled against the absorption spectra under oxy-deoxy-oxy transform are also presented in Table 1. The transfer between the oxygenation- and the deoxygenation-state shows a mixing of the three peaks (540 nm and 575 nm for the oxygenated state, and 553 nm for the deoxygenated state) that can only be seen as a broad “shoulder”. This is due to the fact, that the measurements were performed on a single RBC and the strength of the signal is not sufficient enough to provide a high resolution. However, since the spectra of the purely oxygenated and the deoxygenated states are clear, the result was acceptable.

**Table 1:** **The oxygen content within the channel vs. the time series of the numbered spectra that presented in Figure** 6

**Absorption spectra (No. in Figure ** 6)	**Time (Seconds)**	**O**_**2**_ **-Concentrations (%)**
1	0	17.83 ± 0.14
2	27 ± 0.5	13.030 ± 0.100
3	83 ± 0.5	10.720 ± 0.180
4	128 ± 1	9.607 ± 0.016
5	195 ± 1	6.430 ± 0.030
6	191 ± 1	4.465 ± 0.026
7	227 ± 1	3.481 ± 0.014
8	255 ± 1	2.941 ± 0.018
9	282 ± 1	0.103 ± 0.006
10	298 ± 1	0.017 ± 0.016
11	5400 ± 2	0.023 ± 0.008
12	5828 ± 2	13.650 ± 0.160

The successful tests of the gas-tight functionality within the LOC showed the capability of the LOC to perform electrophysiological measurements on the trapped cell. Long patch pipettes with open-tip resistances of 8-10 M *Ω* were used for sealing onto the RBC plasma membrane. A patch clamp pipette was moved slowly towards the optically trapped cell to ensure near contact between the tip of the pipette and the membrane of the cell. The changes in pipette and access resistances were measured prior to the seal formation. Once the pipette attached the membrane of the cell, the positive pressure was released and followed by the application of gentle suction (negative pressure) on the solution within the pipette to create a high seal resistance. Many RBCs were trapped and patched successfully whereas the highest whole-cell access (Ra) and membrane (Rm) resistances obtained were 5.033 ± 0.412 M *Ω* and 889.7 ± 1.74 M *Ω*. The main focus of this paper was to present a gas-tight LOC that meets all the requirements to enable patch clamp experiment under hypoxic condition. Complete patch clamp experiments on other types of biological cells (related to the projects) during hypoxia will be presented in the future. Compared to our earlier study [42], the LOC was used for experiments under physiological conditions and the viability of the cells was improved by efficient cell transport into the LOC using shorter transport pathways and efficient design of the channel system and enhanced fluid flow.

## Conclusions

In conclusion, we have demonstrated a concept of a low-cost, multi-use and high performance functionality of gas-tight PMMA based LOC for patch clamp investigations on single cells with precise control of hypoxic and anoxic conditions obtained by exchangeable oxygen contents. The LOC presented here provides an alternative to the traditional open system that is conventionally used in patch clamp experiments. The pump systems enabled the best infusion of RBCs and buffer solutions into the microfluidic channels by flow rates lower than 0.5 *μ*l/s. The time series of the absorption spectra showed that the RBCs were viable for an experimental time more than 90 minutes and that the LOC delivered full control over the oxygen content. The measurements of the oxygen content within the LOC as well as the acquired absorption spectra of the patched RBC could to a high grade verify that the chip could be used for hypoxic and normoxic electrophysiological investigations. The resistance measurements of the sealed patch showed the proof of concept that this system could be used in the future to perform a full protocol of patch clamp measurements on different biological cells under controlled environments.

An improvement may be executed such as using absorption spectroscopy to investigate and evaluate the quantitative oxygenation status within the chamber. Another improvement is to incorporate control over temperature or pressure. The presented LOC shows a useful functionality to measure cell mechanics such as sheer stress under environmental changes. Furthermore, the gas-tight functionality and feasible control of the gaseous environments within the chip could be used to provide rapid and direct insight into the behavior and growth of anaerobic microorganisms. The presented LOC may also be useful for studying the effect of changing chemical and physical conditions on the morphology and function of biological cells in environmental gradients.

## References

[1] Sakmann B, Neher E (1984). Patch clamp techniques for studying ionic channels in excitable membranes. Annu Rev Physiol.

[2] Hamill O, Marty A, Neher E, Sakmann B, Sigworth F (1981). Improved patch-clamp techniques for high-resolution current recording from cells and cell-free membrane patches. Pflügers Archiv.

[3] Zhao Y, Inayat S, Dikin D, Ruoff R, Troy J (2009). Impedance characterization and modelling of an improved patch clamp device. Proc Inst Mech Eng, Part N: J Nanoengineering Nanosystems.

[4] Brüggemann A, Farre C, Haarmann C, Haythornthwaite A, Kreir M, Stoelzle S, et al. Planar patch clamp: advances in electrophysiology. In: Potassium Channels. Springer: 2009. p. 165–76. http://www.ncbi.nlm.nih.gov/pubmed/18998092. 10.1007/978-1-59745-526-8_13 18998092

[5] Milligan CJ, Li J, Sukumar P, Majeed Y, Dallas ML, English A (2009). Robotic multiwell planar patch-clamp for native and primary mammalian cells. Nat Protoc.

[6] Park YK, Jung SJ, Yoo J-E, Kwak J, Lim W, Kim J (2003). Effect of acute hypoxia on atp-sensitive potassium currents in substantia gelatinosa neurons of juvenile rats. Pflügers Archiv.

[7] Cheng Y, Gu XQ, Bednarczyk P, Wiedemann FR, Haddad GG, Siemen D (2008). Hypoxia increases activity of the bk-channel in the inner mitochondrial membrane and reduces activity of the permeability transition pore. Cell Physiol Biochem.

[8] Wang L, Greenfield Jr LJ (2009). Post-hypoxic changes in rat cortical neuron gaba A receptor function require l-type voltage-gated calcium channel activation. Neuropharmacology.

[9] Hamann M, Rossi DJ, Mohr C, Andrade AL, Attwell D (2005). The electrical response of cerebellar purkinje neurons to simulated ischaemia. Brain.

[10] Miró M, Hansen EH (2007). Miniaturization of environmental chemical assays in flowing systems: The lab-on-a-valve approach vis-a-vis lab-on-a-chip microfluidic devices. Analytica Chimica Acta.

[11] Schütze K, Pösl H, Lahr G (1998). Laser micromanipulation systems as universal tools in cellular and molecular biology and in medicine. Cell Mol Biol (Noisy-le-Grand, France).

[12] Alrifaiy A, Lindahl OA, Ramser K (2012). Polymer-based microfluidic devices for pharmacy, biology and tissue engineering. Polymers.

[13] Grenz A, Homann D, Eltzschig HK (2011). Extracellular adenosine: a safety signal that dampens hypoxia-induced inflammation during ischemia. Antioxidants Redox Signaling.

[14] Du Y, Lo E, Ali S, Khademhosseini A (2008). Directed assembly of cell-laden microgels for fabrication of 3d tissue constructs. Proc Nat Acad Sci.

[15] Yang C-G, Wu Y-F, Xu Z-R, Wang J-H (2011). A radial microfluidic concentration gradient generator with high-density channels for cell apoptosis assay. Lab Chip.

[16] Fan HC, Wang J, Potanina A, Quake SR (2011). Whole-genome molecular haplotyping of single cells. Nat Biotechnol.

[17] Whitesides GM (2006). The origins and the future of microfluidics. Nature.

[18] Liu W, Li L, Wang X, Ren L, Wang X, Wang J (2010). An integrated microfluidic system for studying cell-microenvironmental interactions versatilely and dynamically. Lab Chip.

[19] Mehta G, Mehta K, Sud D, Song JW, Bersano-Begey T, Futai N (2007). Quantitative measurement and control of oxygen levels in microfluidic poly (dimethylsiloxane) bioreactors during cell culture. Biomed Microdevices.

[20] Berthier E, Warrick J, Casavant B, Beebe DJ (2011). Pipette-friendly laminar flow patterning for cell-based assays. Lab Chip.

[21] Polinkovsky M, Gutierrez E, Levchenko A, Groisman A (2009). Fine temporal control of the medium gas content and acidity and on-chip generation of series of oxygen concentrations for cell cultures. Lab Chip.

[22] Adler M, Polinkovsky M, Gutierrez E, Groisman A (2010). Generation of oxygen gradients with arbitrary shapes in a microfluidic device. Lab Chip.

[23] Thomas PC, Raghavan SR, Forry SP (2011). Regulating oxygen levels in a microfluidic device. Anal Chem.

[24] Skolimowski M, Nielsen MW, Emnéus J, Molin S, Taboryski R, Sternberg C (2010). Microfluidic dissolved oxygen gradient generator biochip as a useful tool in bacterial biofilm studies. Lab Chip.

[25] Chen Y-A, King AD, Shih H-C, Peng C-C, Wu C-Y, Liao W-H (2011). Generation of oxygen gradients in microfluidic devices for cell culture using spatially confined chemical reactions. Lab Chip.

[26] Park J, Bansal T, Pinelis M, Maharbiz MM (2006). A microsystem for sensing and patterning oxidative microgradients during cell culture. Lab Chip.

[27] Brischwein M, Motrescu E, Cabala E, Otto A, Grothe H, Wolf B (2003). Functional cellular assays with multiparametric silicon sensor chips. Lab Chip.

[28] Tourovskaia A, Figueroa-Masot X, Folch A (2005). Differentiation-on-a-chip: a microfluidic platform for long-term cell culture studies. Lab Chip.

[29] Kane BJ, Zinner MJ, Yarmush ML, Toner M (2006). Liver-specific functional studies in a microfluidic array of primary mammalian hepatocytes. Anal Chem.

[30] Wang Z, Kim M-C, Marquez M, Thorsen T (2007). High-density microfluidic arrays for cell cytotoxicity analysis. Lab Chip.

[31] Lam RH, Kim M-C, Thorsen T (2009). Culturing aerobic and anaerobic bacteria and mammalian cells with a microfluidic differential oxygenator. Anal Chem.

[32] Szita N, Boccazzi P, Zhang Z, Boyle P, Sinskey AJ, Jensen KF (2005). Development of a multiplexed microbioreactor system for high-throughput bioprocessing. Lab Chip.

[33] De Bartolo L, Salerno S, Morelli S, Giorno L, Rende M, Memoli B (2006). Long-term maintenance of human hepatocytes in oxygen-permeable membrane bioreactor. Biomaterials.

[34] Sud D, Mehta G, Mehta K, Linderman J, Takayama S, Mycek M-A (2006). Optical imaging in microfluidic bioreactors enables oxygen monitoring for continuous cell culture. J Biomed Opt.

[35] Radisic M, Deen W, Langer R, Vunjak-Novakovic G (2005). Mathematical model of oxygen distribution in engineered cardiac tissue with parallel channel array perfused with culture medium containing oxygen carriers. Am J Physiol-Heart Circulatory Physiol.

[36] Beebe DJ, Mensing GA, Walker GM (2002). Physics and applications of microfluidics in biology. Annu Rev Biomed Eng.

[37] Leclerc E, Sakai Y, Fujii T (2004). Microfluidic pdms (polydimethylsiloxane) bioreactor for large-scale culture of hepatocytes. Biotechnol Progr.

[38] Zhang Z, Boccazzi P, Choi H-G, Perozziello G, Sinskey AJ, Jensen KF (2006). Microchemostat-microbial continuous culture in a polymer-based, instrumented microbioreactor. Lab Chip.

[39] Higgins J, Eddington D, Bhatia S, Mahadevan L (2007). Sickle cell vasoocclusion and rescue in a microfluidic device. Proc Nat Acad Sci.

[40] Houston K, Weinkauf D, Stewart F (2002). Gas transport characteristics of plasma treated poly (dimethylsiloxane) and polyphosphazene membrane materials. J Membr Sci.

[41] Ashkin A, Dziedzic J, Yamane T (1987). Optical trapping and manipulation of single cells using infrared laser beams. Nature.

[42] Alrifaiy A, Ramser K (2011). How to integrate a micropipette into a closed microfluidic system: absorption spectra of an optically trapped erythrocyte. Biomed Opt express.

[43] Ashkin A, Dziedzic J (1987). Optical trapping and manipulation of viruses and bacteria. Science.

[44] Blank M, Kiger L, Thielebein A, Gerlach F, Hankeln T, Marden MC (2011). Oxygen supply from the bird’s eye perspective globin e is a respiratory protein in the chicken retina. J Biol Chem.

[45] Marchand P, Marmet L (1983). Binomial smoothing filter: a way to avoid some pitfalls of least squares polynomial smoothing. Rev Sci Instrum.

[46] Weir EK, Cabrera JA, Mahapatra S, Peterson DA, Hong Z (2010). The role of ion channels in hypoxic pulmonary vasoconstriction. Adv Exp Med Biol.

